# Supplementary material for: Dioscin Alleviates Cisplatin-Induced Mucositis in Rats by Modulating Gut Microbiota, Enhancing Intestinal Barrier Function and Attenuating TLR4/NF-κB Signaling Cascade
Source: Int J Mol Sci. 2022 Apr 17;23(8):4431. doi: 10.3390/ijms23084431 (PMC9025408; doi:10.3390/ijms23084431)
Supplement: Supplementary file 1 [file ijms-23-04431-s001.zip › ijms-1642340-supplementary.pdf]

Supplementary results

Supplementary Table S1.Primer sequences and cycling conditions for real-time. PCR

Gene	Forward 5′ to 3′	Reverse 5′ to 3′
MUC4	ATACATCAACTACATCAGCC	CTCTGAGGAAGTGTTGTTAT
MUC2	GCCAGATCCCGAAACCA	TATAGGAGTCTCGGCAGTCA
ZO-1	CCCTCTGATCATTCCACACA	TTTAGACATGCGCTCTTCCT
Occludin	CTCCAACGGCAAAGTGAATG	CGGACAAGGTCAGAGGAATC
Claudin-1	CTGGCTTCGCTGGGATGGA	TGGCCTGAGCAGTCACGATGTT
IL6	AGGAGTGGCTAAGGACCAAGACC	TGCCGAGTAGACCTCATAGTGACC
IL1β	ATCTCACAGCAGCATCTCGACAAG	CACACTAGCAGGTCGTCATCATCC
TNF α	GCATGATCCGAGATGTGGAACTGG	CGCCACGAGCAGGAATGAGAAG
IL10	TGCCTTCAGCAGAGTGAAG	GGGAAGAAATCGATGACAG
TLR4	AGCCGGAAGGTTATTGTGGTAGT	TGCCGTTTCTTGTTCTTCCTCT
NF-κB	GCATTCTGACCTTGCCTATC	ATCCTTCCCAAACTCCACC
IκBα	TACGCCCCAGCATCTCCACTCCG	CTCCACGATGCCCAGGTAGCCAT
MyD88	GAGCAGTGTCCCACAGACAA	AGTAGCAGATGAAGGCGTCG
GAPDH	TTCAACGGCACAGTCAAGG	CACCAGTGGATGCAGGGA

Supplementary Table S2. Anosim Analysis.

Group	R-value	p-value
CDDP vs Con	0.574	0.002
Dio+CDDP vs Con	0.463	0.005
Dio+CDDP vs CDDP	0.121	0.142

Note: The R-value is between (-1, 1), and the R value is greater than 0, indicating that the difference is significant. The R-value is less than 0, indicating that the difference within the group is greater than the difference, and the reliability of statistical analysis is expressed by the P-value.

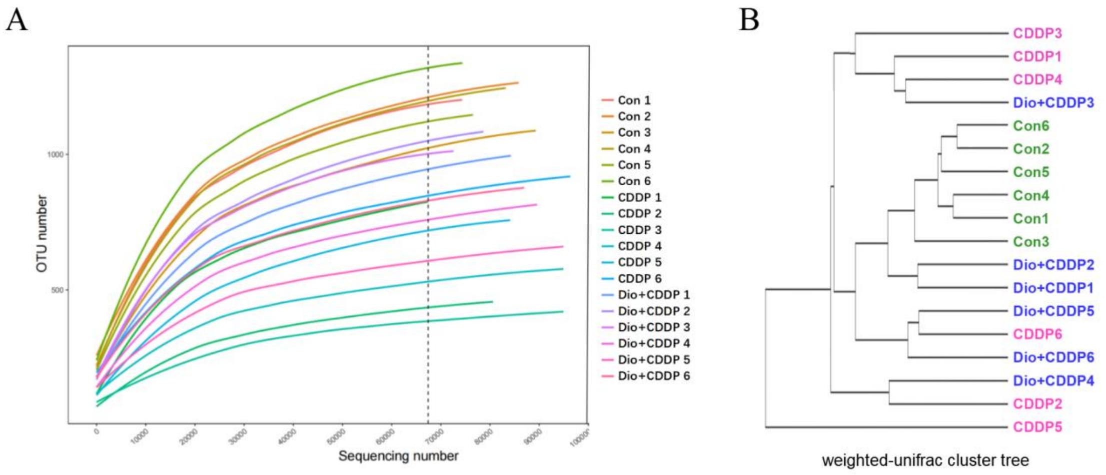

Figure S1. The rarefaction curves of OTU numbers (A), UPGMA clustering tree based on weighted UniFrac distance (B).

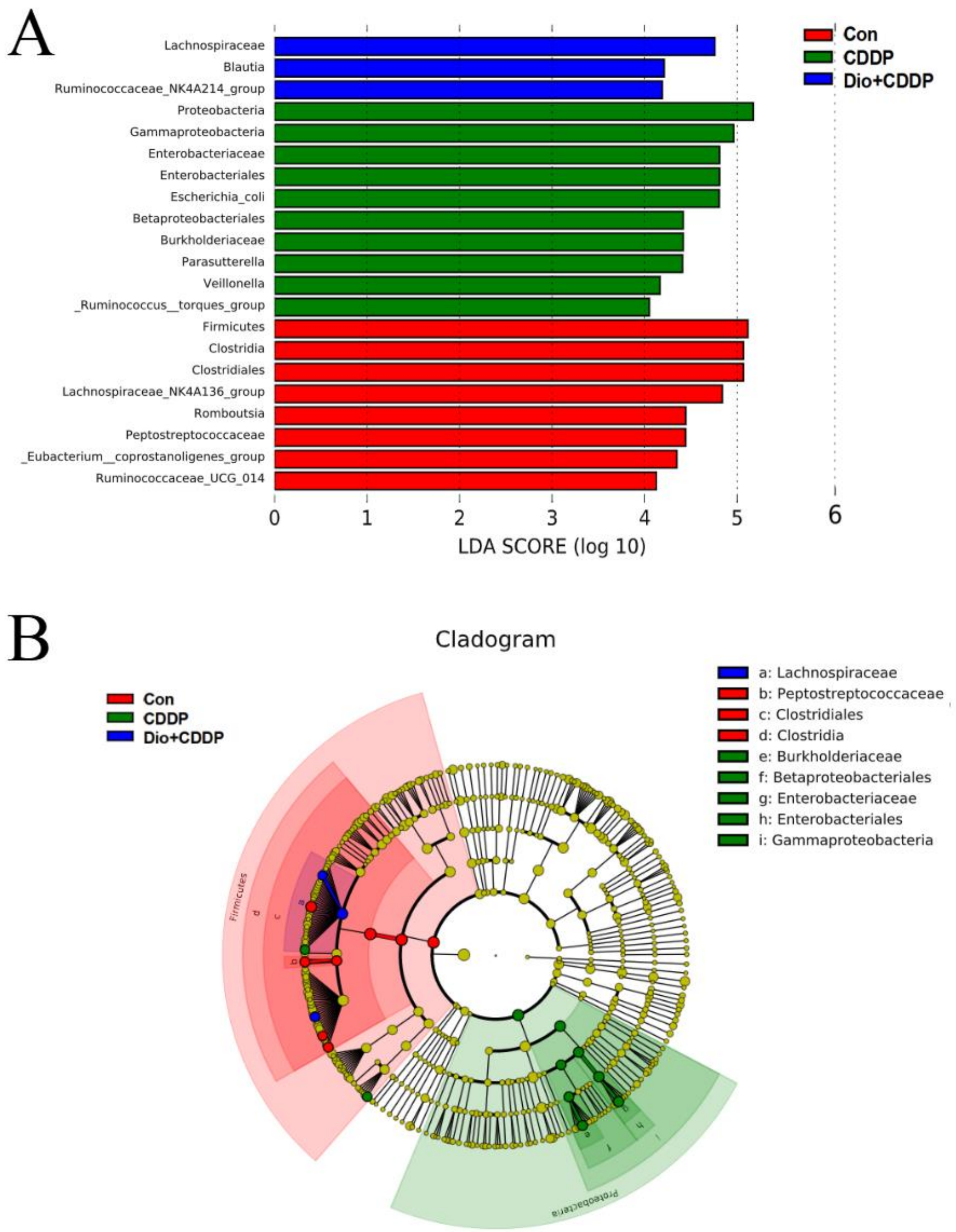

Figure S2. (A) Linear discriminant analysis (LDA). An LDA score higher than 4 indicated the biomarker in the corresponding group has significant different among four groups. An LDA length (LDA score) indicated affect degree of 21 significant species. The taxa with the largest effect sizes (LDA Score >4) are presented. (B) Cladogram (circular hierarchical tree), radial pattern of from inner to outer circular represent from phylum to genus (species) and each dot represents a bacterial taxon at same level. The one innermost dot represent the highest level of taxonomy the kingdoms Bacteria, followed outwards by the lower levels, including phylum, class, order, family and genus. Each circle's diameter is proportional to the taxon's abundance. Each color represents different group.
